# Perceived Knowledge and Attitudes of Faculty Members towards Inclusive Education for Students with Disabilities: Evidence from a Greek University

**DOI:** 10.3390/ijerph19042151

**Published:** 2022-02-14

**Authors:** Maria Papadakaki, Anastasia Maraki, Nikolaos Bitsakos, Joannes Chliaoutakis

**Affiliations:** Department of Social Work, School of Health Sciences, Hellenic Mediterranean University, 71004 Heraklion, Greece; tasiamaraki@gmail.com (A.M.); nbitsakos@hmu.gr (N.B.); jchlia@hmu.gr (J.C.)

**Keywords:** university students, faculty members, educational needs, disability, attitudes, inclusion

## Abstract

The current paper aimed at investigating factors affecting the perceptions and attitudes of faculty members towards inclusive education for students with disabilities in a Greek University. A questionnaire, based on the “Expanding Cultural Awareness of Exceptional Learners-ExCEL” was distributed online to 311 faculty members, during the first semester of 2020. The questionnaire explored participants’ sociodemographic and academic background, prior training and personal experience with disability, perceived knowledge, beliefs and attitudes towards inclusive education practices. A total of 80 questionnaires were completed (males 56.3%; aged 41–50 years 43.7%; working experience > 16 years 52.4%; prior training on disability 77.5%). Factor analysis identified four constructs relevant to: (a) perceived knowledge regarding the legal framework (“Perceived Knowledge”), (b) intention towards the provision of general accommodations in class (“Help in Class”), (c) intention towards resource provision (“Material Offer”), and (d) beliefs about the provision of accommodations to students with disabilities (“Negative Attitude”). Gender, faculty subject and prior training on disability were shown to affect the participants’ “Perceived Knowledge”, while working position was shown to affect “Material Offer”. Age, working experience, and personal experience with disability did not reveal any significant effect. More research is needed to investigate the attitudinal and practical barriers of faculty members towards meeting students’ educational needs.

## 1. Introduction

Individuals with disabilities constitute a large proportion of the general population of higher education students. A recent large-scale survey identified 19.4% of students with disabilities, primarily with mental disorders (35.0%), learning difficulties (33.5%) and chronic diseases (16.1%) [1]. These numbers are probably underestimated, due to many “undiagnosed” students and many others choosing not to disclose their disability, fearing “stigmatization”.

Most importantly, individuals with disabilities have been found to be less likely to enroll in higher education and more likely to experience study delays, while running a higher risk of dropping out of higher education than students without disabilities [2,3,4]. They have also been shown to face several challenges, including discrimination from peers, the lack of an empathetic approach from faculty members, and inadequate accommodations [5,6,7,8].

In order to address these barriers, it is imperative for higher education to ensure an inclusive learning environment, through establishing an inclusive “culture”, “practice”, and “policy” [9]. Regarding “culture”, we primarily expect positive attitudes towards disability from students and staff. By “inclusive practice” we foresee reasonable accommodations to adjust the educational circumstances to the students’ needs. By “inclusive policy” we expect institutional decisions that support the inclusive education model through increasing practices that lead to inclusion and eliminating barriers to exclusion, in a framework based on the principles of justice and equality [10]. 

Several researchers suggest an “anticipatory approach of the curricular design” to achieve “inclusive education”, with a strategy for academic assessment and activities that satisfy the learning needs of every student and promote the social and educational participation of all students [11,12,13,14,15,16]. The most common “frameworks” of inclusive education (Universal Design for Assessment, UDA; Universal Design for Instruction, UDI; Universal Design for Learning, UDL), have been considered to have five themes in common including: (a) backward design or the formulation of learning goals and objectives (e.g., on the course syllabus), (b) multiple means of presentation (e.g., course materials in print formats), (c) inclusive teaching strategies and learner supports (e.g., summarizing key points, small group work), (d) inclusive assessment (e.g., allowing students to use combinations of writing, speaking, and other ways to demonstrate mastery of knowledge), and (e) instructor approachability and empathy (e.g., allowing multiple options for engagement) [17]. 

In line with the principles of inclusive education, it is noted that higher education institutes need to offer various support options, which are designed to address the academic challenges of students with disabilities and ongoing support or coaching [18]. Support modes can either focus on helping students to obtain better functional capabilities (e.g., improving academic skills through workshops on compensatory strategies) or aimed at changing the educational environment so that these students can succeed despite their disabilities (e.g., technological devices, human helpers) [19]. 

Despite these principles, achieving inclusive education still seems problematic. Universities are not proactive in ensuring reasonable adjustments for students with disabilities. They tend to address disability as an “individualized problem” that requires special help, rather than focus on restructuring educational environments so that disabled individuals can be included [20]. Research suggests that faculty is confronted with various obstacles when attempting to make reasonable adjustments. Most importantly, significant gaps have been noted in faculty understanding of disability, as well as misperceptions of the characteristics of students with disabilities [12,20,21]. 

### 1.1. Faculty Attitudes and Inclusive Education 

Faculty judgments and behaviors are often influenced by favorable or unfavorable evaluations of persons and circumstances (attitudes), a process that involves cognitive, affective and behavioral components. This evaluation process is either automatic (activated upon encountering persons or circumstances) or more deliberate (involving careful consideration of consequences of certain behaviors) [22,23,24]. Therefore, one of the greatest challenges in inclusive education, apart from providing faculty with skills, knowledge and understanding, is to ensure the development of positive attitudes toward students with disabilities and their inclusion in regular classrooms [25].

Despite this fact, faculty members have been seen as a source of stigma, whether due to lack of understanding or mistrust of a students’ need for accommodations [5,26]. It is often the case that they do not feel competent to teach students with disabilities in their classroom or they are not committed enough to support students and implement reasonable accommodations in practice [27]. They are often criticized for not making adjustments to the content, the teaching ways and the classroom environment [28]. 

The low competence and commitment of faculty members is often attributed to a lack of training in inclusive education, which disempowers them from taking further action [29,30]. Prior research shows clear relationships between faculty attitudes and prior training and experience [31,32]. Besides training, research indicates a number of personal and professional characteristics as exerting an influential role on faculty members’ attitudes, including age, gender, working position and rank, level of exposure to people with a disability, prevailing beliefs about disability, and others [33,34]. For example, females have been shown to report more positive attitudes toward inclusive education than their male counterparts, be more proactive towards providing accommodations and more likely to identify curricular obstacles that students with disabilities must overcome [35,36]. Likewise, faculty with more years of experience have been shown to be in favor of greater accessibility and university resources for students with disabilities [36,37]. 

Despite the knowledge gained in the last decade regarding the educational barriers for students with disabilities in higher education, there is still a lack of research investigating the link between teachers’ attitudes, self-efficacy and practices [38,39,40]. We still seek reasonable explanations about why students struggle with whether it is worthwhile to disclose their disability as well as why they refrain from making use of the available resources and support [26,39,40,41,42]. Although studies on staff experiences of disabled students have been conducted in Europe, the US and Australia, studies in Greece have focused on school aged students, specifically, those with learning disabilities [43]. There are limited studies in higher education that focus on the faculty perspective. Our study is one of the few that focuses on faculty perceptions and attitudes in the Greek higher education context.

### 1.2. The Greek Situation

In Greece, Law1351/1983 was the first to allow individuals with certain disabilities to be admitted to higher education departments, except those indicated as difficult to attend, due to the nature of the science, according to a reasoned decision of the department, which should be approved by the senate and announced before the beginning of each academic year. “Physically weak” candidates were required to submit an opinion from the Primary Health Committee that they cannot take the written general examinations, due to permanent or temporary, physical or sensory impairment (Presidential Decree 238/1988). Laws 2413/1996 and 2640/1998 introduced new categories of individuals with disabilities among those enrolling in excess in higher education and with ministerial decisions of 2000–2001, individuals “suffering from serious diseases” were identified as having the right to be admitted to higher education institutes at a rate of 3% of the total admissions per academic year. Law 4186/2013 identified individuals with disabilities falling into certain categories as eligible to be admitted at a rate of 5% of the total admissions per academic year. 

With the circular F5/1449/Β3/4-01-2006, it was recommended that higher education institutes should ensure that places and examination procedures are proportional to the needs of people with disabilities, while institutes were instructed to inform the Ministry about the number of individuals with a disability per department and their emerging needs. Regarding the access to educational material, with F/Β’2065/24-10-2007, publishers were obliged to provide the files of the compulsory textbooks of higher education in electronic form, as well as all the books of primary and secondary education.

As for dyslexia, an earlier circular (F142/Β3/7104/19-12-1990) indicated that dyslexic students must be examined orally in the partial and graduate examinations, while law 3699/2008 officially recognized dyslexia as a disability. That is, students who had a formal diagnosis had the opportunity to be examined orally for entry into higher education. 

Despite the legal achievements of the last two decades, in favor of the students with disabilities, Greek higher education is not yet equipped to serve the diverse needs of these individuals. Existing initiatives promoting equal educational opportunities and reasonable adjustments for disabled students are primarily voluntary and usually undertaken by few motivated faculty members, students’ unions, and, less often, funded by the university authorities themselves or co-financed by European Community programs, without direct links to national policies. 

Research on this topic is also scarce in higher education. One of the few large-scale surveys, in 18 higher education institutes in Greece, indicates the magnitude of the problem in Greek universities. Overall, the survey identified 5086 students with disabilities out of 185,627 in total, with the majority presenting a chronic disease (75.9%) and with the vast majority receiving their degree late or dropping out at some point. One of the largest universities in Greece (AUTH) estimated stagnant students with disabilities at 31.3% and graduates with disabilities for the respective periods not exceeding a maximum of 15%. 

### 1.3. Research Objectives 

The current study was designed to develop further understanding about factors affecting faculty members’ perceptions and attitudes towards inclusive education practices for students with disabilities, in a Greek university. Among the study objectives was to investigate the effect of certain sociodemographic and professional characteristics on faculty perceptions and attitudes towards inclusive education practices. More precisely, we hypothesized that female participants of younger age, nonpermanent work positions, with prior training and longer working experience, would manifest higher levels of perceived knowledge regarding the legal framework applying to higher education and more favorable attitudes towards inclusive education for disabled students as compared with participants with different profiles. In the next paragraphs, we first present the research strategy and the methods used in the current study and we then report on participants’ perceptions and attitudes towards inclusive education practices, placing emphasis on personal and work-related characteristics that have been shown to influence these dimensions. At the end of this paper, we discuss our findings against the findings of prior research, and we consider how faculty can profit from these insights and which strategies could build competence and promote positive attitudes among the faculty in order to achieve a more equitable educational system. 

## 2. Material and Methods

### 2.1. Sampling Process

A structured questionnaire was distributed to faculty members and instructors across 15 academic departments of the Hellenic Mediterranean University, one of the three public higher education institutes of Crete region. The questionnaire was distributed online during the first semester of 2020 to all faculty members with up to date personal information employed at the university (*n* = 311). A total of 80 individuals agreed to participate and completed the questionnaires (25.7% response rate). This response rate is consistent with previous online surveys on similar topics [44,45,46]. Participants completed an online consent form prior to participation in the survey.

### 2.2. Research Instrument

The research instrument was based on the “Expanding Cultural Awareness of Exceptional Learners-ExCEL” [17]. A subset of questions from the original 31-item instrument was used to explore attitudinal constructs of interest. The survey was adapted to include four additional items investigating personal experiences with disability (i.e., be a person with disability or have family members or significant others with disability). Items were also modified to reflect the name, the country’s legal framework and the services of the institution where data were collected. The survey inquired about disability in general but offered detailed definitions of the most prevalent disability types encountered in the country’s higher education institutes (i.e., physical, learning, mental health) in accordance with the legal framework. Inclusion of a case definition was specifically aimed at facilitating a common understanding of key concepts of interest among the study participants. The final instrument presented in the following sections consists of a total of 8 items relevant to faculty personal profile and 15 items relevant to perceived knowledge and attitudes towards inclusive education practices. The 8 items explored basic demographic characteristics (gender, age), selected work-related characteristics (faculty subject, position, work experience) and prior experience with disability (training or personal experience). The 15 items explored a variety of areas, including faculty beliefs about accommodations for students with disabilities (e.g., providing educational facilities to students with certified disabilities is unfair as a method for students without disabilities), their perceived knowledge regarding the country’s legal framework and available resources for students with disabilities (e.g., I am sufficiently aware of the legal framework (Law 4186/2013) as it applies to students with disabilities in higher education), and attitudes toward the provision of accommodations in higher education (e.g., I am willing to reduce the total material of my courses for a student with a certified disability even if I did not allow the total material to be reduced for the other students). A six-point Likert scale was applied to acquire responses on the 15 attitudinal items, ranging from “strongly disagree” (1) to “strongly agree” (6). 

### 2.3. Statistical Analysis

The statistical package SPSS v.23 (IBM Corp, Armonk, NY, USA) was used for data analysis [47]. A principal component factor analysis was conducted to explore the underlying factors of faculty members’ and instructors’ attitudes, perceptions and practices towards disabled students. The Kaiser–Meyer–Olkin (KMO) index and the Bartlett’s test of sphericity were employed to indicate whether the correlation matrices were suitable for factor analysis. Eigenvalues were considered when greater than 1.0 and cut-off loadings of 0.60. The Cronbach’s alpha and composite reliability were used to test reliability of the scales. Composite reliability, convergent validity (using the average variance extracted, AVE), discriminant validity (computed by taking the square root of AVE), and heterotrait–monotrait ratio of correlations (HTMT) of the variables/factors were calculated [48]. The Shapiro–Wilk test was used to examine whether scores were normally distributed in the population. Cronbach’s alpha was calculated to assess the reliability of the new scales. Statistically significant relationships were evaluated using student *t*-test and one-way ANOVA.

## 3. Results

### 3.1. Participants’ Profile

Table 1 presents the participants’ personal information and prior experience with disability. In total, 45 (56.3%) males participated in the study, primarily from the Faculties of Health Sciences (*n* = 30, 37.5%) and Engineering (*n* = 24, 30%). Most of them aged 41–50 years (*n* = 35, 43.7%), had a working experience of 16–20 years (*n* = 23, 28.7%) and were occupied on a contractual basis (*n* = 75, 93.7%). The majority of the participants reported prior training on issues relevant to disability (*n* = 62, 77.5%) and more than half of them had a family member with disability (*n* = 42, 52.5%).

### 3.2. Participants’ Perceptions and Attitudes towards Inclusive Education Practices 

Participants’ perceptions and attitudes towards inclusive education practices are presented in Table 2. In particular, more than 1/3 of the participants strongly disagreed with statements indicating sufficient perceived knowledge of the legal framework of special education applying to higher education (e.g., “I am sufficiently aware of the legal framework (Law 4186/2013) as it applies to students with disabilities in higher education”, “I am sufficiently aware of the circular (F5/1449/Β3/4-1-2006) which concerns facilities for students with disabilities”). Approximately half of the participants strongly agreed with statements indicating willingness to offer assisting material to students with disability (e.g., “I am willing to provide copies of my slides or presentations (power point) to students with certified disabilities”, “I am willing to allow students with certified disabilities to record my course sessions”). Nearly half of the participants strongly disagreed with the statement “I am willing to reduce the total material of my courses for a student with a certified disability even if I did not allow the total material to be reduced for the other students” (*n* = 35, 43.8%). Approximately 1/3 of the participants strongly disagreed with statements indicating negative beliefs about provision of accommodations for students with disabilities (e.g., “I believe that students with disabilities use their disability as an excuse when they are not doing well in my classes”, “At times, I feel overwhelmed when my students with disabilities approach me with requests for facilitation”).

### 3.3. Factor Analysis and Scale Reliability 

The Kaiser–Meyer–Olkin (KMO) index (0.743) and Bartlett’s test of sphericity (χ² = 2028.329, *p* < 0.001) indicated that correlation matrices were suitable for factor analysis. Factor analysis of the ExCEL tool resulted in four factors explaining 69% of variance (“Perceived Knowledge”, “Help in Class”, “Material Offer”, “Negative Attitude”). New variables were composed into clusters using items that showed the highest total correlation for each of the hypothetical constructs we intended to measure, as follows: (i) Perceived Knowledge (five items, mean value 3.0) measured the perceived knowledge regarding the country’s legal framework and available resources for students with disabilities, (ii) “Help in Class” (four items, mean value 3.0) measured the intention towards the provision of general accommodations in class, (iii) “Material Offer” (three items, 5.0) measured the intention towards resource provision, and, (iv) “Negative Attitude” (three items, mean value 2.4) measured beliefs about the provision of accommodations to students with disabilities. Cronbach’s alpha showed that the reliability score was high for the three factors (“Perceived Knowledge”: α = 0.894, “Help in Class”: α = 0.810, “Material Offer”: α = 0.826) and appropriate for “Negative Attitude” (α = 0.609). In addition, the values of composite reliability varied from 0.781 to 0.922, which were all higher than the acceptable minimum of 0.70 [49]. Average variance extracted (AVE) was adopted to examine convergent validity, which represents the extent to which the items of latent variables are theoretically relevant to each other [50]. The AVE values of constructs ranged from 0.546 to 0.707, which are higher than the recommended lowest value of 0.50. The descriptives and the structure of the four new factors are presented in Table 3 and Table 4. Table 5 shows the square root of AVE in a diagonal line, which is greater than the correlation between a pair of latent variables, meeting the standard of discriminant validity [51]. Through examining these indicators, the measurement model had a satisfactory level of reliability and validity. Table 6 shows that the HTMT values have not exceeded the 0.9 thresholds, so it can be concluded the discriminant validity has been established among all constructs [52].

### 3.4. Factors Affecting Perceptions and Attitudes towards Inclusive Education Practices 

In order to identify factors that influence the participants’ perceived knowledge and attitudes towards the provision of accommodations to students with disabilities, we explored the effect of the participants’ basic demographic characteristics (gender, age), selected work-related characteristics (faculty subject, position, work experience) and prior experience with disability (training or personal experience) on the four composite factors (see Table 7).

According to the results, gender, faculty subject and prior training on disability were shown to affect the participants’ “Perceived Knowledge” while the working position was shown to affect “Material Offer”. In particular, gender was shown to be associated at a statistically significant level with “Perceived Knowledge” (t(80) = 3.981, *p* = 0.004), with women shown to have higher perceived knowledge about the legal framework that applies to students with disability in higher education, as compared with men. Similarly, statistically significant associations were identified in terms of the faculty subject and “Perceived Knowledge”, with participants from the Health Sciences demonstrating higher perceived knowledge as compared with their counterparts from Engineering (mean scores 3.4 and 2.5, respectively). Likewise, participants reporting prior training on disability were found to have a higher “Perceived Knowledge” as compared with those not reporting a similar training experience (mean scores 3.7 and 2.7, respectively).

Working position was related at a statistically significant level with “Material Offer” (t(80) = 1.230, *p* = 0.013) with participants holding a nonpermanent working position found to be more willing to provide material to students with disabilities as compared with those holding a permanent working position.

Age, working experience, and prior personal experience with disability did not reveal a statistically significant relationship with any of the composite variables (“Perceived Knowledge”, “Help in Class”, “Material Offer”, “Negative Attitude”).

Overall, the current findings partly verify our initial hypothesis. More precisely, among the sociodemographic characteristics, gender was shown to affect “Perceived Knowledge” but this was not the case for age. Likewise, in terms of work related characteristics, only faculty subject and work position were shown to exert an influential role on “Perceived Knowledge” and “Material Offer,” respectively, but a similar effect was not evident for working experience. Lastly, in regard to prior exposure to disability issues, only prior training was shown to have a significant effect on “Perceived Knowledge”, while personal experience with disability did not demonstrate a similar effect. In addition, despite our initial expectations, perceived attitudes towards inclusive education, and particularly “Help in Class”, “Material Offer” and “Negative Attitudes,” were only associated with the participants’ work position. 

## 4. Discussion

Our results indicate that most of the participants do not feel confident about their knowledge of the legal framework applying to higher education regarding students with disability. Although they seem to be willing to offer material to students with disability, they seem unwilling to modify the teaching procedures and hold unfavorable attitudes about adopting different approaches to meet the diverse needs of students. Previous research identified a lack of knowledge and negative perceptions among faculty members [53,54,55], as well as low awareness of existing disability legislation in higher education [56]. The results further indicate that faculty members have numerous doubts about how to make adjustments and they need more support to carry them out. Many participants perceived that offering support to students with disabilities would create avenues for exploitation. Some participants conceptualized inclusion as not just making individual adjustments, but also “bringing everybody on the same higher education journey, to have equal opportunities to access learning.” Similar misperceptions are evident elsewhere [12]. In some studies, there are misperceptions among faculty members connecting academic adjustments with reduced academic standards [44], while in other studies, faculty members consider the favorable treatment for students with disabilities as unfair treatment for students without disabilities [56]. This could potentially be addressed through introducing targeted training for university staff as well as a coherent national policy for students with disabilities in higher education, which would take into account the individual needs of this population group in the program coordination and the design of curricula. 

What is also important among the study findings, is the fact that women presented higher “Perceived Knowledge” of the legal framework, as compared with men. In line with our findings, previous research identified female tutors as having a more favorable attitude towards people with disabilities than male tutors, as well as making more rigorous attempts to minimize educational barriers as compared with their male colleagues [53,57,58]. This could be attributed to higher levels of empathy found in women as compared with men or to the nurturing and caring roles traditionally assigned to them.

Most importantly, our study identified those members who received prior training on disability as having higher “Perceived Knowledge” as compared with those lacking a similar experience. Previous research has identified the valuable effect of prior training on the faculty awareness and emphasized the need for targeted training of university tutors on issues related to the various types of disability [59]. 

Our results further indicate that faculty members of Health Sciences manifest higher “Perceived Knowledge” as compared to those of other fields. Similarly, Schoen [60] identified that tutors in the fields of humanities were more aware and had more positive views towards students with disability than tutors in the fields of natural and formal sciences. Other studies have also found that tutors in humanities departments are more willing to provide facilities and guidance to students with disability [55,61,62].

Interestingly, faculty members holding a nonpermanent working position were found to be more willing to provide assisting material to students with disabilities as compared with those holding a permanent working position. Other studies have concluded similar findings with nonpermanent teachers reporting greater flexibility in adapting course tasks and requirements, minimizing learning barriers, and providing teaching materials in greater variety than permanent teaching staff [17].

In our study, age, working experience, and prior experience with the disability of a family member did not reveal statistically significant links with faculty members’ attitudes and current practices. This contradicts other studies, which have shown that the older the teachers, the more negative their attitudes regarding inclusion, indicating that while teachers gain professional seniority their attitudes towards inclusion are dampened [63]. In contrast to our findings, other studies also indicate that contact with people with disabilities and previous experience of teaching students with disabilities are associated with teachers’ attitudes towards inclusion [59,64].

### 4.1. Study Limitations

The present study has certain limitations. First, the study was conducted in a single university, and we cannot generalize the results to other higher education institutes of the country. Second, the small sample suggests that the findings cannot be generalized to the general population of faculty members. Third, most of the participants in the sample held a nonpermanent working position, which suggests that the findings could differ if the synthesis of the sample was different. Fourth, the small sample did not allow for more specialized analysis and predictive models to be conducted. Lastly, the method of self-report and retrospective data collection cannot exclude a possible social desirability responding error. 

### 4.2. Suggestions for Future Research

Future research with complementary methods could improve our understanding of the differential effects of attitudes on the adoption of inclusive education practices and the cognitive processes involved in faculty members’ interactions with students with disabilities. The effects of attitude–behavior processes on the implementation of inclusive education practices should be prioritized in future efforts. More information is also needed on the actual behaviors of faculty members and further investigation of students’ perceptions of faculty attitudes and behaviors would be highly valued. Faculty intention to advocate on students’ behalf, and the actual provision of accommodations, should be investigated through students’ reports and verified through alternative research methodologies. Qualitative investigations that more directly document interactions between faculty members and students with disabilities would help to verify the findings of the current study. Review and analysis of evidence regarding students’ visits to university psychosocial and medical services would further demonstrate the actual needs of students and indicate how these needs could be addressed with the support of faculty members and through the necessary policy reforms. 

## 5. Conclusions

The current study reports on very interesting findings regarding faculty member perceptions and attitudes towards inclusive education practices. On one hand, we identified low confidence levels in using such practices, while on the other hand, we discovered high levels of willingness to offer at some extent practical support, which implies “a great potential for improvement”. What stands out among the results is the fact that some faculty are under aware of policies and procedures relevant for students with disabilities, as well as holding misconceptions regarding specific services offered on campus. Likewise, a high level of uncertainty was evident about how to ensure equal opportunities without being unfair to students without disabilities. Equality among our participants seems to translate into “one size strategies for all”, regardless of disabilities. What also seems interesting is the effort to safeguard their credibility and prevent a potentially favorable treatment to those not deserving it. It could be the low awareness of existing legal frameworks combined with high levels of perceived liability that make our participants less flexible in approving accommodations for students with disabilities. Most importantly, the study identified certain characteristics that distinguish those more favorable towards inclusive education against others who are more skeptical. These findings are highly valued, as they could guide future policy by indicating the groups most in need of training and those with the highest potential in bringing changes in terms of inclusive education. These findings are also important because they offer initial evidence on the underlying social norms and cultural beliefs that influence faculty behavior, which could translate into targeted interventions. Such interventions could include onsite training programs, workshops, research initiatives, technological aids and library resources, with an emphasis placed on knowledge, beliefs and behavior change [55,65,66,67,68]. Our participants could also benefit from continuing education and in service courses and, particularly, from clear guidance on how to successfully deliver inclusive education. It is very encouraging that our university has recently established a “special office” for disability and “faculty counsellors” for students with disability, which are considered to be highly valued policy developments towards improving access to services for student with disabilities and towards the promotion of equal opportunities. Moreover, our University Counseling Center, which was established recently as part of the Operational Program “Human Resources Development, Education and Lifelong Learning”, is operated by a multidisciplinary team of professionals (including psychologists, social workers, special education tutors, medical doctors and nurses), and already serves as a point of reference for faculty members for matters of disability. This center offers timely support to students with disabilities and referral to internal or external services, while also promoting awareness on disability within the academic community. Most importantly, since last year, our university has developed an “Observatory of vulnerable students”, which systematically collects important information from various university services, on social circumstances, health and mental health morbidity and comorbidity, accessibility, service utilization, upon the students’ consent. This observatory is the first of its kind across Greek universities and serves as a mechanism of epidemiological surveillance of students’ needs, which offers important evidence to university authorities on the effectiveness of existing interventions and recommendations for policy reforms. Nevertheless, despite the promising developments of the last few years, the current study strongly emphasizes the need to invest more efforts in faculty preparedness, especially in terms of addressing low participant awareness of the legal framework and low confidence in adopting inclusive education practices, as well as tackling negative attitudes and breaking down barriers that prevent students with disabilities from being offered tailor made opportunities to knowledge.

## Figures and Tables

**Table 1:** Demographic and work-related information of the participants.

*n* = 80		F	%
Gender	Male	45	56.3
	Female	35	43.7
Age group	31–40	8	10
	41–50	35	43.7
	51–60	26	32.5
	>61	11	13.7
Work position	Permanent	5	6.3
	Temporary (contract)	75	93.7
Work experience	0–5	12	15
	6–10	5	6.2
	11–15	11	13.7
	16–20	23	28.7
	>21	19	23.7
Faculty subject	Engineering	24	30
	Health Sciences	30	37.5
	Agriculture	8	10
	Management and Economic Science	14	17.5
	Music & Optoacoustic Technologies	4	5
Prior training on disability	Yes	18	22.5
	No	62	77.5
Personal disability	Yes	10	12.5
	No	70	87.5
Disability of family member	Yes	42	52.5
	No	38	47.5

**Table 2:** Participants’ distribution based on their perceptions and attitudes towards inclusive education practices (strongly disagree = 1 to strongly agree = 6).

*n* (%)	1	2	3	4	5	6
I am sufficiently aware of the exact legal definition of disability (Law 3699/2008 ‘‘Special Education and Training of Persons with Disabilities or Special Educational Needs’’)	24 (30.0%)	19 (23.8%)	12 (15.0%)	11 (13.8%)	10 (12.5%)	4 (5.0%)
I am sufficiently aware of the legal framework (Law 4186/2013) as it applies to students with disabilities in higher education.	26 (32.5%)	16 (20.0%)	12 (15.0%)	11 (13.8%)	12 (15.0%)	3 (3.8%)
I am sufficiently aware of the circular (F5/1449/Β3/4-1-2006) that concerns facilities for students with disabilities.	26 (32.5%)	17 (21.3%)	11 (13.8%)	13 (16.3%)	9 (11.3%)	4 (5.0%)
At this stage I do not have sufficient knowledge to provide the appropriate facilities to students with disabilities in my courses.	5 (6.3%)	8 (10.0%)	8 (10.0%)	23 (28.8%)	22 (27.5%)	14 (17.5%)
I know the assistive technology that students with disabilities can use to help understand my course material.	17 (21.3%)	22 (27.5%)	17 (21.3%)	10 (12.5%)	10 (12.5%)	4 (5.0%)
I provide individual facilities to students who have revealed their disability to me.	6 (7.5%)	3 (3.8%)	11 (13.8%)	13 (16.3%)	25 (31.5%)	22 (27.5%)
I am willing to allow a student with a disability to complete extra credits for academic success even when this option is not listed on the curriculum.	11 (13.8%)	5 (6.3%)	14 (17.5%)	20 (25.0%)	15 (18.8%)	15 (18.8%)
I am willing to allow any student to complete extra credits on my courses.	16 (20.0%)	8 (10.0%)	19 (23.8%)	16 (20.0%)	10 (12.5%)	11 (13.8%)
I am willing to reduce the total material of my courses for a student with a certified disability even if I did not allow the total material to be reduced for the other students.	35 (43.8%)	11 (13.8%)	13 (16.3%)	9 (11.3%)	7 (8.8%)	5 (6.3%)
I am willing to provide copies of my lecture notes or course outlines to students with certified disabilities.	1 (1.3%)	1 (1.3%)	4 (5.0%)	10 (12.5%)	19 (23.8%)	45 (56.3%)
I am willing to provide copies of my slides or presentations (power point) to students with certified disabilities.	0 (0%)	2 (2.5%)	5 (6.3%)	9 (11.3%)	21 (26.3%)	43 (53.8%)
I am willing to allow students with certified disabilities to record my course sessions.	4 (5.0%)	5 (6.3%)	7 (8.8%)	10 (12.5%)	15 (18.8%)	39 (48.8%)
Providing educational facilities to students with certified disabilities is unfair as a method for students without disabilities.	38 (47.5%)	17 (21.3%)	14 (17.5%)	7 (8.8%)	3 (3.8%)	1 (1.3%)
I believe that students with disabilities use their disability as an excuse when they are not doing well in my classes.	23 (28.8%)	21 (26.3%)	17 (21.3%)	11 (13.8%)	6 (7.5%)	2 (2.5%)
At times, I feel overwhelmed when my students with disabilities approach me with requests for facilitation.	27 (33.8%)	13 (16.3%)	17 (21.3%)	11 (13.8%)	8 (10.0%)	4 (5.0%)

**Table 3:** Factor analysis results.

	Component			
**Factor 1: Perceived Knowledge**	1	2	3	4
I am sufficiently aware of the exact legal definition of disability (Law 3699/2008 ‘‘Special Education and Training of Persons with Disabilities or Special Educational Needs’’)	0.897	0.02	−0.046	0.042
I am sufficiently aware of the legal framework (Law 4186/2013) as it applies to students with disabilities in higher education.	0.949	−0.066	−0.022	0.076
I am sufficiently aware of the circular (Φ5/1449/Β3/ 4-1-2006) which concerns facilities for students with disabilities.	0.916	−0.045	−0.018	0.035
At this stage I do not have sufficient knowledge to provide the appropriate facilities to students with disabilities in my courses.	0.651	0.036	0.052	0.037
I know the assistive technology that students with disabilities can use to help understand my course material.	0.751	0.065	0.034	0.056
**Factor 2: Help in Class**				
I provide individual facilities to students who have revealed their disability to me.	−0.293	0.661	0.119	0.064
I am willing to allow a student with a disability to complete extra credits for academic success even when this option is not listed on the curriculum.	0.13	0.818	0.107	0.101
I am willing to allow any student to complete extra credits on my courses.	0.029	0.899	0.144	0.024
I am willing to reduce the total material of my courses for a student with a certified disability even if I did not allow the total material to be reduced for the other students.	0.088	0.768	0.125	−0.076
**Factor 3: Material Offer**				
I am willing to provide copies of my lecture notes or course outlines to students with certified disabilities.	−0.137	0.136	0.86	0.188
I am willing to provide copies of my slides or presentations (power point) to students with certified disabilities.	−0.021	0.123	0.902	0.188
I am willing to allow students with certified disabilities to record my course sessions.	0.168	0.222	0.741	−0.037
**Factor 4: Negative Attitude**				
Providing educational facilities to students with certified disabilities is unfair as a method for students without disabilities.	0.021	0.015	0.229	0.768
I believe that students with disabilities use their disability as an excuse when they are not doing well in my classes.	0.191	0.018	−0.109	0.791
At times, I feel overwhelmed when my students with disabilities approach me with requests for facilitation.	−0.005	0.039	0.151	0.649
Cronbach’s alpha	0.894	0.81	0.826	0.609
Composite reliability	0.922	0.869	0.875	0.781
AVE	0.707	0.625	0.701	0.546
Extraction Method: Principal Component Analysis, Rotation Method: Varimax with Kaiser Normalization.				

**Table 4:** Participants’ mean scores in the four factors.

Factor	*n* (%)	Mean	SD
Perceived Knowledge	80	3	0.9
Help in Class	80	3	1
Material Offer	80	5	1
Negative Attitude	80	2.4	1

**Table 5:** Discriminant validity analysis results.

Construct	SumKnowledge	SumPositiveHelpClass	SumPositiveHelpMaterial	SumNegativeAttitude
SumKnowledge	0.841			
SumPositiveHelpClass	−0.019	0.791		
SumPositiveHelpMaterial	−0.001	0.297 **	0.837	
SumNegativeAttitude	−0.126	0.165	−0.233 *	0.738

* *p* < 0.05, ** *p* < 0.01.

**Table 6:** Heterotrait-Monotrait ratio (HTMT).

Construct	SumKnowledge	SumPositiveHelpClass	SumPositiveHelpMaterial	SumNegativeAttitude
SumKnowledge				
SumPositiveHelpClass	0.182			
SumPositiveHelpMaterial	0.138	0.412		
SumNegativeAttitude	0.199	0.144	0.361	

**Table 7:** Factors affecting perceptions and attitudes towards inclusive education practices.

Mean (SD)	Factor 1: Perceived Knowledge	Factor 2: Help in Class	Factor 3: Material Offer	Factor 4: NegativeAttitude
Gender	0.004 **	0.878	0.795	0.316
*Male*	2.7 (0.9)	3.0 (1.1)	5.1 (1.0)	2.5 (1.1)
*Female*	3.3 (1.0)	3.0 (1.0)	5.0 (1.0)	2.2 (0.8)
Age groups	0.312	0.574	0.654	0.797
*31–40*	2.5 (0.5)	3.4 (0.9)	5.0 (1.0)	2.1 (1.1)
*41–50*	3.2 (1.0)	2.8 (1.2)	4.9 (1.1)	2.4 (1.0)
*51–60*	2.8 (0.9)	3.1 (0.8)	5.2 (0.8)	2.4 (0.9)
*>61*	3.0 (1.1)	3.0 (1.0)	5.1 (0.9)	2.2 (1.2)
Work position	0.823	0.783	0.013 *	0.58
*Permanent*	3.0 (1.0)	3.0 (1.0)	5.0 (1.0)	2.3 (1.0)
*Temporary (Contract)*	2.9 (0.8)	3.2 (1.1)	5.8 (0.4)	2.8 (1.5)
Work experience	0.326	0.342	0.363	0.771
*0–5*	2.7 (0.9)	3.4 (1.0)	5.1 (1.0)	2.2(0.7)
*6–10*	3.4 (1.0)	3.1 (1.4)	5.2 (0.7)	2.6 (1.5)
*11–15*	3.2 (1.2)	3.2 (1.4)	5.4 (0.9)	2.1 (1.0)
*16–20*	3.2 (0.8)	2.6 (1.0)	4.7 (1.1)	2.5 (1.0)
*>21*	2.7 (1.0)	3.1 (0.9)	5.1 (0.9)	2.4 (1.0)
Faculty subject	0.011 *	0.16	0.933	0.475
*Engineering*	2.5 (0.7)	3.3 (0.8)	5.1 (1.1)	2.6 (1.1)
*Health Sciences*	3.4 (1.0)	3.0 (1.1)	5.1 (0.9)	2.3 (1.0)
*Agriculture*	2.4 (0.8)	3.0 (1.2)	4.7 (1.4)	2.4 (0.8)
*Management and Economic Science*	3.1 (1.1)	2.4 (1.1)	5.0 (1.0)	2.2 (1.0)
*Music & Optoacoustic Technologies*	3.1 (0.7)	3.3 (0.6)	5.1 (0.8)	1.8 (0.6)
Prior training on disability	0.001 **	0.479	0.922	0.059
*Yes*	3.7 (0.9)	2.8 (1.2)	5.1 (0.9)	2.0 (0.7)
*No*	2.7 (0.9)	3.1 (1.0)	5.0 (1.0)	2.5 (1.0)
Own disability	0.798	0.708	0.948	0.384
*Yes*	2.9 (1.4)	3.1 (1.0)	5.0 (1.3)	2.1 (0.8)
*No*	3.0 (0.9)	3.0 (1.1)	5.0 (1.0)	2.4 (1.0)
Disability of family member	0.17	0.59	0.752	0.884
*Yes*	3.1 (1.0)	3.1 (1.1)	5.1 (1.0)	2.4 (0.9)
*No*	2.8 (0.9)	2.9 (1.0)	5.0 (1.0)	2.3 (1.1)

* *p* < 0.05, ** *p* < 0.01.

## Data Availability

Data available upon request.
